# Synthesis and crystal structure of [Sr(urea)(NO_3_)_2_]_*n*_

**DOI:** 10.1107/S2056989024012386

**Published:** 2025-01-07

**Authors:** Aysanem Bektursinova, Zulfiya Djumanazarova, Zamira Uzakbergenova, Jamshid Ashurov, Akram A Khan, Shakhnoza Kadirova, Batirbay Torambetov

**Affiliations:** a Karakalpak State University, 1 Ch. Abdirov St Nukus, 230112, Uzbekistan; b Institute of Bioorganic Chemistry, Academy of Sciences of Uzbekistan, M. Ulugbek St, 83, Tashkent, 100125, Uzbekistan; c Physical and Materials Chemistry Division CSIR-National Chemical Laboratory,Pune-411008 India; d National University of Uzbekistan named after Mirzo Ulugbek 4 University St Tashkent 100174 Uzbekistan; University of Neuchâtel, Switzerland

**Keywords:** crystal structure, strontium, caramid, urea, Hirshfeld surface analysis

## Abstract

The crystal structure of poly[di-μ_2_-nitrato-μ_2_-urea-strontium(II)], [Sr(NO_3_)_2_(CH_4_N_2_O)]_*n*_, was determined using single-crystal X-ray diffraction. Crystallizing in the ortho­rhom­bic space group *Aba*2, the asymmetric unit consists of an Sr^II^ cation, two nitrate anions, and two half urea mol­ecules. The Sr^II^ cation adopts a distorted deca­hedral geometry coordinated by ten oxygen atoms, with Sr—O bond lengths ranging from 2.573 (3) to 2.847 (5) Å. The nitrate anions act as bidentate ligands, displaying both terminal and bridging coordination modes. The structure features a robust coordination network supported by hydrogen bonding. These results provide insight into the coord­ination behaviour of Sr^II^ with nitrate and urea ligands, contributing to the understanding of supra­molecular architectures in metal–organic frameworks (MOFs).

## Abstract 2

The mol­ecular and crystal structure of the [Sr(urea)(NO_3_)_2_]_*n*_ complex was studied to investigate the various inter­molecular inter­actions.

## 1. Chemical context

The study of coordination polymers (CPs) and the crystal engineering of MOFs has garnered significant inter­est due to their diverse structural architectures and potential applications in catalysis, gas storage, and sensing (Allendorf & Stavila, 2015 ▸). Metal ions such as Sr^II^ have proven versatile in forming coordination complexes owing to their ability to adopt various coordination geometries (Kainat *et al.*, 2024 ▸). Ligands like urea and nitrate, capable of acting as terminal and bridging ligands, offer unique opportunities for the construction of supra­mol­ecular networks (Reek *et al.*, 2022 ▸). In this study, the polymeric complex [Sr(urea)(NO_3_)_2_]_*n*_ was synthesized and characterized.
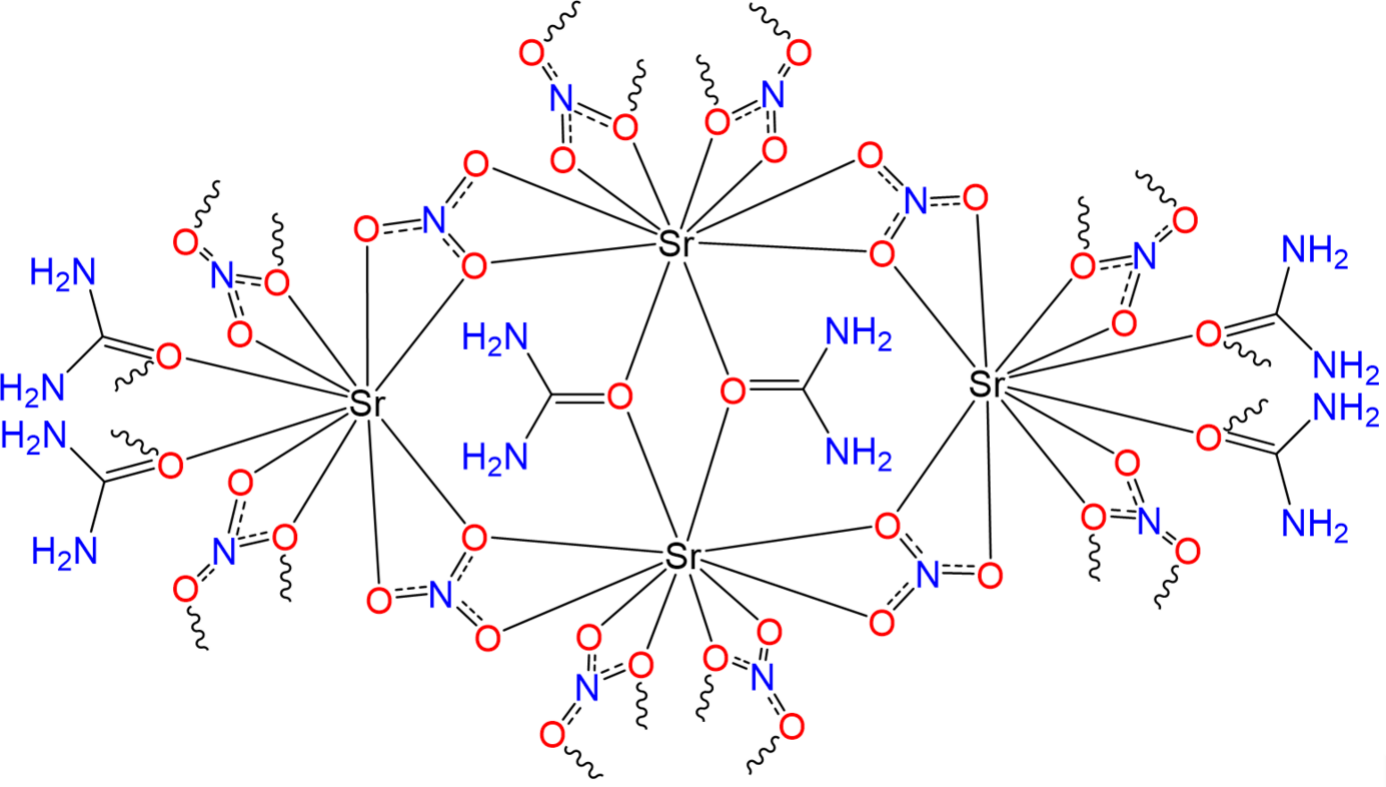


## 2. Structural commentary

**Figure 1:**
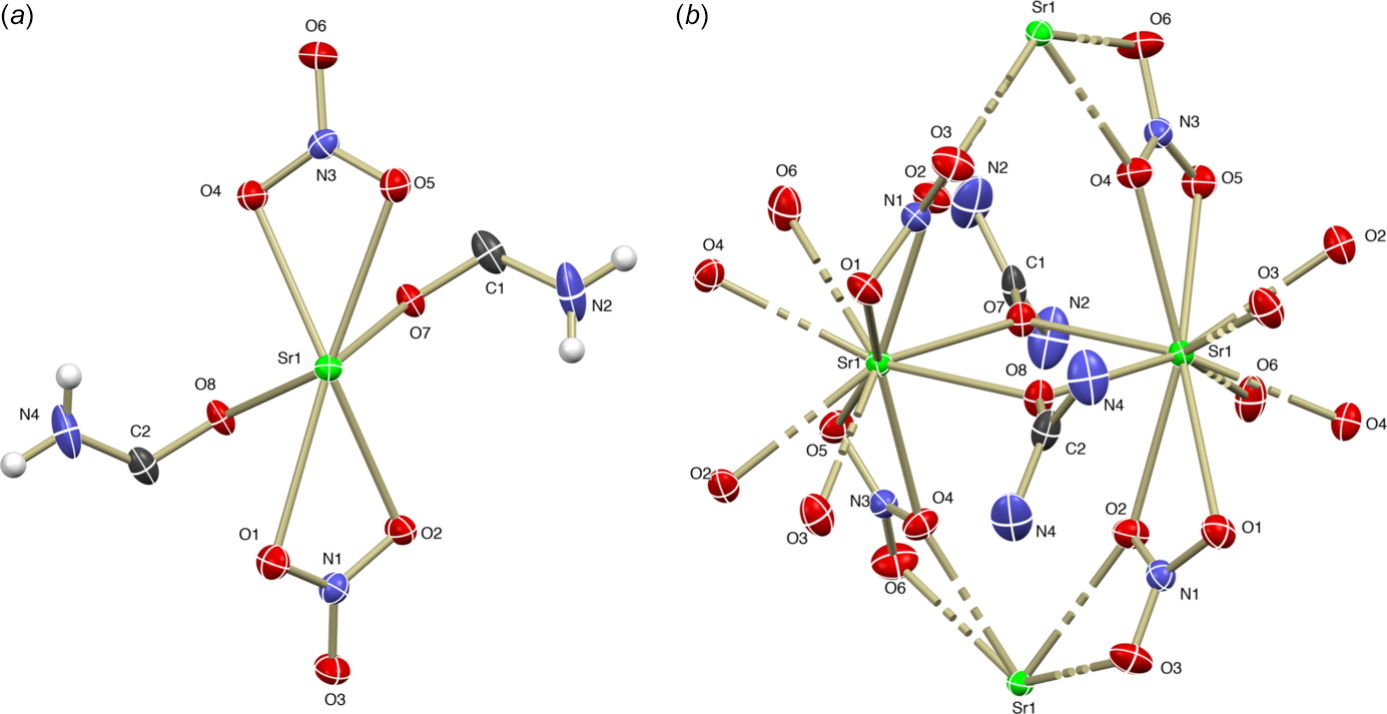
(*a*) The asymmetric unit of [Sr(urea)(NO_3_)_2_]_*n*_ with the atom-labelling scheme and displacement ellipsoids drawn at the 30% probability level. (*b*) Extended coordination sphere of the polymeric complex.

The crystal structure of the title compound [Sr(urea)(NO_3_)_2_]_*n*_ was determined in the ortho­rhom­bic space group *Aba*2. The asymmetric unit consists of an Sr^II^ cation, two nitrate anions, and one urea mol­ecule. The Sr^II^ cation is coordinated by ten oxygen atoms, eight of which originate from nitrate anions and two from urea mol­ecules, adopting a deca­hedral geometry (Fig. 1 ▸). The Sr^II^ cation forms a distorted deca­hedral coordination environment through inter­actions with oxygen atoms from the urea and nitrate ligands. The nitrate anions exhibit dual binding modes, participating in bidentate bridging inter­actions, which stabilize the structure through hydrogen bonds and inter­mol­ecular forces. Such versatility in coordination and binding contributes to the robust di-periodic layered network observed in the crystal structure. These findings provide insights into the design of Sr^II^-based CPs and MOFs, highlighting the significance of urea and nitrate ligands in generating diverse structural motifs and functional materials (Preethi *et al.*, 2024 ▸).

## 3. Supra­molecular features

**Figure 2:**
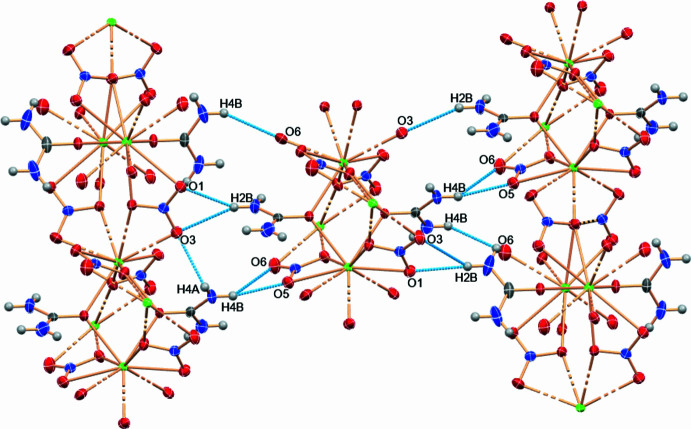
Hydrogen-bonded chains (blue dashed lines) in the crystal of [Sr(urea)(NO_3_)_2_]_*n*_.

In the crystal, closely associated mol­ecules form a di-periodic sheet structure along the *a*- and *b*-axis directions. Along the *c* axis, mol­ecules are connected by hydrogen bonds (N4—H4*A*⋯O3^ii^, N4—H4*B*⋯O5^vi^, N4—H4*B*⋯O6^vi^, N2—H2*A*⋯O6^i^, N2—H2*B*⋯O1^vii^, N2—H2*B*⋯O3^vii^; Table 1 ▸). The Sr—O (Sr-nitrate) bond lengths range from 2.622 (3) Å to 2.847 (5) Å, while the Sr–O (Sr-urea) bond lengths fall between 2.573 (3) Å and 2.604 (3) Å, reflecting variations due to ligand-field effects and steric factors (Fig. 1 ▸). The nitrate anions act as bidentate ligand, contributing to the coordination geometry in two distinct modes. First, two oxygen atoms from each nitrate mol­ecule coordinate to the same Sr^II^ ion. Second, the oxygen atoms O7 and O8 are bidentate bridging ligands, connecting two Sr^II^ cations and forming a distorted parallelogram. The bond angles of the bridging oxygen atoms are 108.0 (2)° for (Sr—O7—Sr) and 106.13 (19)° for (Sr—O8—Sr). In the crystal structure, the urea mol­ecules are located on a special position with a twofold rotation axis at (−*x*, −*y*, *z*), oriented along the [001] direction. This structural arrangement results in a stable coordination network supported by inter­actions between the Sr^II^ cations, nitrate anions, and urea (Fig. 2 ▸).

## 4. Database survey

A survey of the Cambridge Structural Database (CSD, Version 5.45, last updated March 2024; Groom *et al.*, 2016 ▸) revealed around 320 metal complexes where urea is directly bonded to a metal *via* oxygen, whereas only one structure where Sr is directly bonded to the oxygen atom of the urea mol­ecule has been reported (MOXJUG; Schwarz & Streb, 2015 ▸). Moreover, no crystal structure similar to that of [Sr(urea)(NO_3_)_2_]_*n*_ has been reported.

## 5. Synthesis and crystallization

Strontium nitrate (Sr(NO_3_)_2_, 0.212 g, 1 mmol) and carbamide (urea, 0.12 g, 2 mmol) were each individually dissolved in 5 mL of a 1:1 volumetric mixture of water and ethanol, ensuring complete dissolution of both compounds. The solutions were mixed together and kept for 10 min in an ultrasonic bath. The obtained colourless solution was filtered and left for crystallization. Single crystals of the title complex suitable for X-ray analysis were obtained by slow evaporation of the solution over a period of 10 days.

## 6. Refinement

Crystal data, data collection and structure refinement details are summarized in Table 2 ▸. All hydrogen atoms were located in difference-Fourier maps and reﬁned using an isotropic approximation.

## Supplementary Material

Crystal structure: contains datablock(s) I. DOI: 10.1107/S2056989024012386/tx2092sup1.cif

CCDC reference: 2297418

Additional supporting information:  crystallographic information; 3D view; checkCIF report

## Figures and Tables

**Table 1:** Hydrogen-bond geometry (Å, °)

*D*—H⋯*A*	*D*—H	H⋯*A*	*D*⋯*A*	*D*—H⋯*A*
N4—H4*A*⋯O3^i^	0.86	2.44	2.956 (6)	119
N4—H4*B*⋯O5^ii^	0.86	2.29	3.086 (6)	154
N4—H4*B*⋯O6^ii^	0.86	2.59	3.304 (7)	141
N2—H2*A*⋯O6^iii^	0.86	2.50	3.020 (6)	120
N2—H2*B*⋯O1^iv^	0.86	2.33	3.139 (7)	157
N2—H2*B*⋯O3^iv^	0.86	2.57	3.312 (6)	145

Symmetry codes: (i) 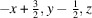; (ii) 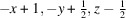; (iii) 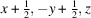; (iv) 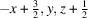.

**Table 2:** Experimental details

Crystal data	
Chemical formula	[Sr(NO_3_)_2_(CH_4_N_2_O)]
*M*_r_	271.70
Crystal system, space group	Orthorhombic, *Aba*2
Temperature (K)	293
*a*, *b*, *c* (Å)	9.3527 (1), 9.9701 (1), 17.0496 (2)
*V* (Å^3^)	1589.83 (3)
*Z*	8
Radiation type	Cu *K*α
μ (mm^−1^)	9.77
Crystal size (mm)	0.12 × 0.08 × 0.06

Data collection	
Diffractometer	XtaLAB Synergy, Single source at home/near, HyPix3000
Absorption correction	Multi-scan (*CrysAlis PRO*; Rigaku OD, 2022 ▸)
*T*_min_, *T*_max_	0.300, 1.000
No. of measured, independent and observed [*I* > 2σ(*I*)] reflections	6435, 1517, 1487
*R*_int_	0.029
(sin θ/λ)_max_ (Å^−1^)	0.614

Refinement	
*R*[*F*^2^ > 2σ(*F*^2^)], *wR*(*F*^2^), *S*	0.021, 0.055, 1.07
No. of reflections	1517
No. of parameters	121
No. of restraints	1
H-atom treatment	H-atom parameters constrained
Δρ_max_, Δρ_min_ (e Å^−3^)	0.74, −0.27
Absolute structure	Flack *x*)’ determined using 678 quotients [(*I*^+^)−(*I*^−^)]/[(*I*^+^)+(*I*^−^)] (Parsons *et al.*, 2013 ▸)
Absolute structure parameter	−0.020 (15)

Computer programs: *CrysAlis PRO* (Rigaku OD, 2022 ▸), *SHELXT2018/2* (Sheldrick, 2015*a* ▸), *SHELXL2016/6* (Sheldrick, 2015*b* ▸) and *OLEX2* (Dolomanov *et al.*, 2009 ▸).

## supplementary crystallographic information

### Ooly[di-µ2-nitrato-µ2-urea-strontium(II)] Crystal data

**Table:**

[Sr(NO_3_)_2_(CH_4_N_2_O)]	*D*_x_ = 2.270 Mg m^−^^3^
*M**_r_* = 271.70	Cu *K*α radiation, λ = 1.54184 Å
Orthorhombic, *A**b**a*2	Cell parameters from 5341 reflections
*a* = 9.3527 (1) Å	θ = 4.7–71.3°
*b* = 9.9701 (1) Å	µ = 9.77 mm^−^^1^
*c* = 17.0496 (2) Å	*T* = 293 K
*V* = 1589.83 (3) Å^3^	Block, colourless
*Z* = 8	0.12 × 0.08 × 0.06 mm
*F*(000) = 1056	

### Ooly[di-µ2-nitrato-µ2-urea-strontium(II)] Data collection

**Table:**

XtaLAB Synergy, Single source at home/near, HyPix3000 diffractometer	1517 independent reflections
Radiation source: micro-focus sealed X-ray tube, PhotonJet (Cu) X-ray Source	1487 reflections with *I* > 2σ(*I*)
Mirror monochromator	*R*_int_ = 0.029
Detector resolution: 10.0000 pixels mm^-1^	θ_max_ = 71.2°, θ_min_ = 5.2°
ω scans	*h* = −11→10
Absorption correction: multi-scan (CrysAlisPro; Rigaku OD, 2022)	*k* = −11→12
*T*_min_ = 0.300, *T*_max_ = 1.000	*l* = −20→20
6435 measured reflections	

### Ooly[di-µ2-nitrato-µ2-urea-strontium(II)] Refinement

**Table:**

Refinement on *F*^2^	H-atom parameters constrained
Least-squares matrix: full	*w* = 1/[σ^2^(*F*_o_^2^) + (0.0374*P*)^2^ + 0.1494*P*] where *P* = (*F*_o_^2^ + 2*F*_c_^2^)/3
*R*[*F*^2^ > 2σ(*F*^2^)] = 0.021	(Δ/σ)_max_ = 0.001
*wR*(*F*^2^) = 0.055	Δρ_max_ = 0.74 e Å^−^^3^
*S* = 1.07	Δρ_min_ = −0.27 e Å^−^^3^
1517 reflections	Extinction correction: *SHELXL2016/6* (Sheldrick, 2015*b*), Fc^*^=kFc[1+0.001xFc^2^λ^3^/sin(2θ)]^-1/4^
121 parameters	Extinction coefficient: 0.00038 (6)
1 restraint	Absolute structure: Flack *x*)' determined using 678 quotients [(*I*^+^)-(*I*^-^)]/[(*I*^+^)+(*I*^-^)] (Parsons *et al.*, 2013)
Hydrogen site location: inferred from neighbouring sites	Absolute structure parameter: −0.020 (15)

### Ooly[di-µ2-nitrato-µ2-urea-strontium(II)] Special details

**Table:**

Geometry. All esds (except the esd in the dihedral angle between two l.s. planes) are estimated using the full covariance matrix. The cell esds are taken into account individually in the estimation of esds in distances, angles and torsion angles; correlations between esds in cell parameters are only used when they are defined by crystal symmetry. An approximate (isotropic) treatment of cell esds is used for estimating esds involving l.s. planes.

### Ooly[di-µ2-nitrato-µ2-urea-strontium(II)] Fractional atomic coordinates and isotropic or equivalent isotropic displacement parameters (Å^2^)

**Table:**

	*x*	*y*	*z*	*U*_iso_*/*U*_eq_	
Sr1	0.66197 (3)	0.35686 (3)	0.50071 (5)	0.02435 (15)	
O5	0.5691 (4)	0.2029 (4)	0.6237 (2)	0.0380 (8)	
O7	0.500000	0.500000	0.5894 (3)	0.0317 (12)	
O4	0.4300 (3)	0.2135 (4)	0.52232 (18)	0.0361 (8)	
O8	0.500000	0.500000	0.4089 (3)	0.0328 (12)	
O1	0.8252 (4)	0.4683 (4)	0.3800 (2)	0.0396 (9)	
O2	0.7896 (4)	0.5891 (3)	0.48352 (18)	0.0367 (8)	
O6	0.3592 (4)	0.1112 (5)	0.6265 (3)	0.0508 (10)	
O3	0.8632 (4)	0.6835 (4)	0.3775 (2)	0.0463 (9)	
C1	0.500000	0.500000	0.6641 (5)	0.042 (2)	
N3	0.4530 (4)	0.1754 (4)	0.5924 (2)	0.0314 (8)	
N4	0.4670 (6)	0.3903 (6)	0.2952 (3)	0.0610 (14)	
H4A	0.445171	0.317865	0.319881	0.073*	
H4B	0.467264	0.391253	0.244752	0.073*	
N2	0.6152 (6)	0.5375 (8)	0.7034 (3)	0.0719 (18)	
H2A	0.690719	0.562025	0.678455	0.086*	
H2B	0.614571	0.537260	0.753809	0.086*	
N1	0.8270 (4)	0.5799 (4)	0.4124 (3)	0.0312 (9)	
C2	0.500000	0.500000	0.3348 (5)	0.038 (2)	

### Ooly[di-µ2-nitrato-µ2-urea-strontium(II)] Atomic displacement parameters (Å^2^)

**Table:**

	*U*^11^	*U*^22^	*U*^33^	*U*^12^	*U*^13^	*U*^23^
Sr1	0.0243 (2)	0.0232 (2)	0.0255 (2)	0.00128 (10)	0.0001 (2)	0.00073 (18)
O5	0.0368 (19)	0.0425 (19)	0.0346 (18)	−0.0014 (15)	−0.0072 (14)	0.0028 (15)
O7	0.037 (3)	0.036 (3)	0.022 (3)	0.006 (2)	0.000	0.000
O4	0.0326 (15)	0.0439 (18)	0.032 (2)	−0.0061 (14)	−0.0059 (11)	0.0078 (13)
O8	0.037 (3)	0.043 (3)	0.019 (3)	0.011 (2)	0.000	0.000
O1	0.049 (2)	0.031 (2)	0.038 (2)	−0.0005 (14)	0.0039 (14)	−0.0070 (15)
O2	0.0462 (16)	0.0334 (16)	0.030 (2)	−0.0066 (13)	0.0083 (14)	−0.0046 (12)
O6	0.051 (2)	0.059 (2)	0.043 (2)	−0.0199 (18)	0.0080 (18)	0.010 (2)
O3	0.065 (2)	0.037 (2)	0.036 (2)	−0.0119 (19)	0.0026 (19)	0.0080 (17)
C1	0.054 (5)	0.042 (5)	0.029 (5)	0.028 (4)	0.000	0.000
N3	0.029 (2)	0.0276 (18)	0.037 (2)	0.0002 (15)	0.0008 (16)	−0.0026 (16)
N4	0.084 (4)	0.072 (3)	0.028 (2)	0.021 (3)	−0.013 (3)	−0.016 (2)
N2	0.070 (4)	0.110 (5)	0.036 (3)	0.030 (4)	−0.021 (3)	−0.024 (3)
N1	0.0311 (19)	0.029 (2)	0.034 (2)	0.0006 (14)	−0.0019 (14)	−0.0016 (17)
C2	0.034 (4)	0.056 (6)	0.025 (5)	0.015 (4)	0.000	0.000

### Ooly[di-µ2-nitrato-µ2-urea-strontium(II)] Geometric parameters (Å, º)

**Table:**

Sr1—O5	2.740 (4)	O4—N3	1.272 (5)
Sr1—O7	2.573 (3)	O8—C2	1.264 (10)
Sr1—O4	2.624 (3)	O1—N1	1.243 (6)
Sr1—O4^i^	2.629 (3)	O2—N1	1.265 (5)
Sr1—O8	2.604 (3)	O6—N3	1.232 (5)
Sr1—O1	2.793 (4)	O3—N1	1.239 (6)
Sr1—O2^ii^	2.723 (3)	C1—N2	1.322 (7)
Sr1—O2	2.622 (3)	C1—N2^iii^	1.322 (7)
Sr1—O6^i^	2.847 (5)	N4—H4A	0.8600
Sr1—O3^ii^	2.731 (4)	N4—H4B	0.8600
Sr1—N3	3.088 (4)	N4—C2	1.322 (7)
Sr1—N1	3.097 (4)	N2—H2A	0.8600
O5—N3	1.241 (5)	N2—H2B	0.8600
O7—C1	1.274 (11)		
			
O5—Sr1—O1	163.75 (10)	O2^ii^—Sr1—N1	124.65 (11)
O5—Sr1—O6^i^	72.03 (13)	O6^i^—Sr1—N3	95.41 (13)
O5—Sr1—N3	23.62 (10)	O6^i^—Sr1—N1	87.87 (13)
O5—Sr1—N1	158.76 (11)	O3^ii^—Sr1—O5	101.94 (11)
O7—Sr1—O5	71.00 (11)	O3^ii^—Sr1—O1	74.45 (12)
O7—Sr1—O4^i^	128.84 (10)	O3^ii^—Sr1—O6^i^	134.94 (11)
O7—Sr1—O4	74.49 (8)	O3^ii^—Sr1—N3	87.94 (12)
O7—Sr1—O8	72.94 (8)	O3^ii^—Sr1—N1	97.10 (12)
O7—Sr1—O1	122.30 (10)	N3—Sr1—N1	169.13 (10)
O7—Sr1—O2	81.01 (8)	N3—O5—Sr1	94.1 (3)
O7—Sr1—O2^ii^	134.83 (9)	Sr1—O7—Sr1^iii^	108.0 (2)
O7—Sr1—O6^i^	82.92 (12)	C1—O7—Sr1	126.00 (10)
O7—Sr1—O3^ii^	138.81 (11)	C1—O7—Sr1^iii^	126.00 (10)
O7—Sr1—N3	69.81 (9)	Sr1—O4—Sr1^iv^	156.08 (14)
O7—Sr1—N1	100.42 (9)	N3—O4—Sr1	98.9 (2)
O4—Sr1—O5	47.58 (9)	N3—O4—Sr1^iv^	102.3 (2)
O4^i^—Sr1—O5	92.60 (10)	Sr1—O8—Sr1^iii^	106.13 (19)
O4—Sr1—O4^i^	128.56 (4)	C2—O8—Sr1	126.94 (9)
O4—Sr1—O1	140.54 (10)	C2—O8—Sr1^iii^	126.94 (9)
O4^i^—Sr1—O1	71.84 (10)	N1—O1—Sr1	92.1 (3)
O4—Sr1—O2^ii^	67.60 (11)	Sr1—O2—Sr1^v^	158.35 (14)
O4^i^—Sr1—O2^ii^	66.10 (11)	N1—O2—Sr1	99.7 (2)
O4^i^—Sr1—O6^i^	46.09 (10)	N1—O2—Sr1^v^	97.4 (2)
O4—Sr1—O6^i^	119.40 (13)	N3—O6—Sr1^iv^	92.7 (3)
O4^i^—Sr1—O3^ii^	91.21 (11)	N1—O3—Sr1^v^	97.7 (3)
O4—Sr1—O3^ii^	72.06 (12)	O7—C1—N2	120.4 (4)
O4—Sr1—N3	24.02 (9)	O7—C1—N2^iii^	120.4 (4)
O4^i^—Sr1—N3	112.09 (11)	N2^iii^—C1—N2	119.2 (8)
O4—Sr1—N1	150.61 (10)	Sr1—N3—Sr1^iv^	110.78 (12)
O4^i^—Sr1—N1	77.56 (10)	O5—N3—Sr1^iv^	172.2 (3)
O8—Sr1—O5	125.63 (9)	O5—N3—Sr1	62.2 (2)
O8—Sr1—O4	84.36 (7)	O5—N3—O4	119.1 (4)
O8—Sr1—O4^i^	141.75 (10)	O4—N3—Sr1^iv^	54.49 (19)
O8—Sr1—O1	69.95 (10)	O4—N3—Sr1	57.1 (2)
O8—Sr1—O2	73.36 (8)	O6—N3—Sr1	173.7 (4)
O8—Sr1—O2^ii^	124.70 (9)	O6—N3—Sr1^iv^	64.3 (3)
O8—Sr1—O6^i^	140.21 (11)	O6—N3—O5	122.4 (4)
O8—Sr1—O3^ii^	80.46 (12)	O6—N3—O4	118.5 (4)
O8—Sr1—N3	104.90 (9)	H4A—N4—H4B	120.0
O8—Sr1—N1	66.70 (8)	C2—N4—H4A	120.0
O1—Sr1—O6^i^	99.01 (12)	C2—N4—H4B	120.0
O1—Sr1—N3	162.17 (11)	C1—N2—H2A	120.0
O1—Sr1—N1	23.64 (11)	C1—N2—H2B	120.0
O2^ii^—Sr1—O5	65.54 (10)	H2A—N2—H2B	120.0
O2—Sr1—O5	136.45 (11)	Sr1—N1—Sr1^v^	114.45 (13)
O2—Sr1—O4^i^	79.48 (11)	O1—N1—Sr1^v^	177.6 (3)
O2—Sr1—O4	150.83 (11)	O1—N1—Sr1	64.3 (2)
O2—Sr1—O1	46.95 (10)	O1—N1—O2	119.2 (4)
O2^ii^—Sr1—O1	102.69 (11)	O2—N1—Sr1^v^	59.1 (2)
O2—Sr1—O2^ii^	141.08 (5)	O2—N1—Sr1	56.5 (2)
O2^ii^—Sr1—O6^i^	94.76 (12)	O3—N1—Sr1	165.5 (3)
O2—Sr1—O6^i^	71.96 (14)	O3—N1—Sr1^v^	59.3 (3)
O2—Sr1—O3^ii^	120.82 (11)	O3—N1—O1	122.4 (5)
O2^ii^—Sr1—O3^ii^	46.46 (10)	O3—N1—O2	118.4 (4)
O2^ii^—Sr1—N3	65.51 (11)	O8—C2—N4^iii^	120.7 (4)
O2—Sr1—N3	149.57 (10)	O8—C2—N4	120.7 (4)
O2—Sr1—N1	23.74 (10)	N4—C2—N4^iii^	118.6 (8)
			
Sr1—O5—N3—O4	5.5 (4)	Sr1—O8—C2—N4^iii^	−117.5 (3)
Sr1—O5—N3—O6	−175.1 (4)	Sr1—O1—N1—O2	14.2 (4)
Sr1^iii^—O7—C1—N2	−117.6 (4)	Sr1—O1—N1—O3	−165.2 (4)
Sr1^iii^—O7—C1—N2^iii^	62.4 (4)	Sr1^v^—O2—N1—Sr1	−166.7 (2)
Sr1—O7—C1—N2	62.4 (4)	Sr1—O2—N1—Sr1^v^	166.7 (2)
Sr1—O7—C1—N2^iii^	−117.6 (4)	Sr1—O2—N1—O1	−15.3 (4)
Sr1^iv^—O4—N3—Sr1	−168.9 (2)	Sr1^v^—O2—N1—O1	178.0 (3)
Sr1—O4—N3—Sr1^iv^	168.9 (2)	Sr1—O2—N1—O3	164.1 (3)
Sr1—O4—N3—O5	−5.8 (4)	Sr1^v^—O2—N1—O3	−2.6 (4)
Sr1^iv^—O4—N3—O5	−174.7 (3)	Sr1^iv^—O6—N3—O5	175.3 (4)
Sr1^iv^—O4—N3—O6	5.9 (5)	Sr1^iv^—O6—N3—O4	−5.3 (4)
Sr1—O4—N3—O6	174.8 (4)	Sr1^v^—O3—N1—Sr1	68.3 (14)
Sr1^iii^—O8—C2—N4^iii^	62.5 (3)	Sr1^v^—O3—N1—O1	−178.0 (3)
Sr1—O8—C2—N4	62.5 (3)	Sr1^v^—O3—N1—O2	2.6 (4)
Sr1^iii^—O8—C2—N4	−117.5 (3)		

Symmetry codes: (i) *x*+1/2, −*y*+1/2, *z*; (ii) −*x*+3/2, *y*−1/2, *z*; (iii) −*x*+1, −*y*+1, *z*; (iv) *x*−1/2, −*y*+1/2, *z*; (v) −*x*+3/2, *y*+1/2, *z*.

### Ooly[di-µ2-nitrato-µ2-urea-strontium(II)] Hydrogen-bond geometry (Å, º)

**Table:**

*D*—H···*A*	*D*—H	H···*A*	*D*···*A*	*D*—H···*A*
N4—H4*A*···O3^ii^	0.86	2.44	2.956 (6)	119
N4—H4*B*···O5^vi^	0.86	2.29	3.086 (6)	154
N4—H4*B*···O6^vi^	0.86	2.59	3.304 (7)	141
N2—H2*A*···O6^i^	0.86	2.50	3.020 (6)	120
N2—H2*B*···O1^vii^	0.86	2.33	3.139 (7)	157
N2—H2*B*···O3^vii^	0.86	2.57	3.312 (6)	145

Symmetry codes: (i) *x*+1/2, −*y*+1/2, *z*; (ii) −*x*+3/2, *y*−1/2, *z*; (vi) −*x*+1, −*y*+1/2, *z*−1/2; (vii) −*x*+3/2, *y*, *z*+1/2.
